# Blasticidin-S deaminase, a new selection marker for genetic transformation of the diatom *Phaeodactylum tricornutum*

**DOI:** 10.7717/peerj.5884

**Published:** 2018-11-14

**Authors:** Jochen M. Buck, Carolina Río Bártulos, Ansgar Gruber, Peter G. Kroth

**Affiliations:** 1 Department of Biology, University of Konstanz, Konstanz, Germany; 2 Institute of Parasitology, Biology Centre, Czech Academy of Sciences, České Budějovice, Czech Republic

**Keywords:** *Phaeodactylum tricornutum*, Voriconazole, Genetic transformation, Genome editing, Tunicamycin, Resistance gene, Blasticidin-S, Selection marker, Antibiotics

## Abstract

Most genetic transformation protocols for the model diatom *Phaeodactylum tricornutum* rely on one of two available antibiotics as selection markers: Zeocin (a formulation of phleomycin D1) or nourseothricin. This limits the number of possible consecutive genetic transformations that can be performed. In order to expand the biotechnological possibilities for *P. tricornutum*, we searched for additional antibiotics and corresponding resistance genes that might be suitable for use with this diatom. Among the three different antibiotics tested in this study, blasticidin-S and tunicamycin turned out to be lethal to wild-type cells at low concentrations, while voriconazole had no detectable effect on *P. tricornutum*. Testing the respective resistance genes, we found that the blasticidin-S deaminase gene (*bsr*) effectively conferred resistance against blasticidin-S to *P. tricornutum*. Furthermore, we could show that expression of *bsr* did not lead to cross-resistances against Zeocin or nourseothricin, and that genetically transformed cell lines with resistance against Zeocin or nourseothricin were not resistant against blasticidin-S. In a proof of concept, we also successfully generated double resistant (against blasticidin-S and nourseothricin) *P. tricornutum* cell lines by co-delivering the *bsr* vector with a vector conferring nourseothricin resistance to wild-type cells.

## Introduction

*Phaeodactylum tricornutum* is one of the most widely used models in diatom research. The genome of *P. tricornutum* is completely sequenced (Bowler et al., 2008) and different methods for the introduction of transgenes are available, for example, particle bombardment (Apt, Grossman & Kroth-Pancic, 1996), conjugation with bacteria (Karas et al., 2015), or electroporation (Miyahara et al., 2013; Niu et al., 2012; Zhang & Hu, 2014). In addition, genome editing methods like TALEN or CRISPR have been introduced recently, allowing the direct knockout of specific genes (Daboussi et al., 2014; Gruber & Kroth, 2017; Nymark et al., 2016; Serif et al., 2017; Slattery et al., 2018). Despite these important achievements, the availability of antibiotics and the number of selection markers for diatoms is very low (compiled in Huang & Daboussi (2017)). Essentially two antibiotics are widely used for the selection of transformed diatom cells, both of which were introduced more than two decades ago: phleomycin (e.g., Zeocin/phleomycin D1), together with the respective *Sh*Ble resistance gene, as well as nourseothricin and the *nat*/*sat*-1 genes (Apt, Grossman & Kroth-Pancic, 1996; Falciatore et al., 1999; Huang & Daboussi, 2017; Sakaguchi, Nakajima & Matsuda, 2011; Zaslavskaia et al., 2000). There are two more pairs of antibiotics and resistance genes that have been reported, but that are not widely used: The antibiotic G418/neomycin and the resistance gene *npt*II have been used by Zaslavskaia et al. (2000), however, the antibiotic is only effective at low salt concentrations (Apt, Grossman & Kroth-Pancic, 1996; Zaslavskaia et al., 2000), and strains expressing *npt*II reach only limited resistance to G418 (Zaslavskaia et al., 2000). Furthermore, the chloramphenicol acetyltransferase was previously published as a reporter gene by Apt, Grossman & Kroth-Pancic (1996), and more recently used by Niu et al. (2012) as a resistance marker for the selection of genetically transformed cell lines on chloramphenicol. Other antibiotics commonly used in protists, like hygromycin and puromycin, were tested in *P. tricornutum*, but either proved ineffective (hygromycin) or only effective at very high concentrations (e.g. puromycin) (Apt, Grossman & Kroth-Pancic, 1996).

The lack of selection markers currently limits genetic modifications of diatoms to a maximum of two independent (or consecutive) screening steps. This is a limiting factor in biotechnological applications, because already the knockout of a single gene via the TALEN system requires two antibiotic resistance markers to select for the presence of the two (left and right) required TALEN constructs (Serif et al., 2017). Further steps like complementation or introduction of a second gene knockout therefore are difficult to achieve. While transient genetic transformations and protein delivery might offer workarounds to this limitation in the future (Karas et al., 2015; Serif et al., 2018; Slattery et al., 2018), additional selection markers will nonetheless be needed whenever stable genome integration of transgenes is required.

Phleomycins target and cleave the DNA of eukaryotic as well as prokaryotic organisms, ultimately resulting in cell death (Berdy, 1980; Maeda et al., 1956). In human cell lines, the expression of the resistance gene *Sh*Ble does not completely inhibit DNA cleavage and unpredictable mutations still occur (Trastoy, Defais & Larminat, 2005). Such phenomena have not yet been observed in *P. tricornutum*, however, particularly for genome editing, it appears essential to use antibiotics that do not affect non-targeted DNA.

In this study, we tested the toxicity of three antibiotics, tunicamycin, blasticidin-S and voriconazole to *P. tricornutum* cells. Only the *N*-glycosylation inhibitor tunicamycin and the protein synthesis inhibitor blasticidin-S effectively caused cell death of *P. tricornutum*. For blasticidin-S, we were able to establish the appropriate resistance gene blasticidin-S deaminase (*bsr*) as selection marker in *P. tricornutum*. We further showed that the *bsr* confers resistance to blasticidin-S only and does not alter the effectiveness of Zeocin or nourseothricin.

## Materials and Methods

### *Phaeodactylum tricornutum* culture media

One liter of modified f/2 medium (Guillard, 1975) was prepared by dissolving 16.6 or 8.3 g of “Tropic-Marin CLASSIC” artificial sea salt (Dr. Biener GmbH, Wartenberg, Germany) in demineralized water (resulting in one half or one quarter of the salinity of natural sea water). After autoclaving the standard f/2 supplements (Guillard, 1975) and additionally one ml of 2M Tris–HCl pH 8.0 were added. For preparation of solid media, 12 g bacto agar (Becton; Dickinson and Company, Le Pont de Claix, France) per liter of salt water were added prior to autoclaving. After addition of the supplements, plates each containing 25 ml of solid medium were prepared in Petri dishes (633181; Greiner Bio-One, Kremsmünster, Austria).

### *Phaeodactylum tricornutum* selection media

Zeocin (Thermo Fisher, Waltham, MA, USA): 750 μl of a 100 mg/ml solution of Zeocin was added to one liter of the f/2 medium to a final concentration of 75 μg/ml. Nourseothricin (clonNAT; WERNER BioAgents, Jena, Germany) was prepared as a stock solution of 100 mg/ml in water and added to the f/2 medium a final concentration of 150 μg/ml. Blasticidin-S (R21001; Thermo Fisher, Waltham, MA, USA) and tunicamycin (Abcam, Cambridge, UK) were prepared as stock solutions of 10 mg/ml in water. Zeocin, nourseothricin, blasticidin-S and tunicamycin stock solutions were added in the desired quantities to the media after autoclaving and prior to preparing the culture plates, or prior to the inoculation in case of liquid selection media. Voriconazole (Sigma-Aldrich, St. Louis, MO, USA) was dissolved in dimethyl sulfoxide as stock solution of 20 mg/ml. Each voriconazole plate was prepared by plating the appropriate amount of the stock solution onto culture plates prepared as described above.

### Transformation of *P. tricornutum* by particle bombardment

Genetic transformation of *P. tricornutum* Pt4 (single colony derived from UTEX646 which was previously used by Apt, Grossman & Kroth-Pancic (1996); UTEX culture collection of algae, Austin, TX, USA) was performed as previously described (Apt, Grossman & Kroth-Pancic, 1996; Kroth, 2007) with three single ballistic shots for one attempt. Selection on blasticidin-S plates was performed at 70 μE (Biolux L 36/965; Osram, Munich, Germany) continuous light conditions and at 22 °C for 3–5 weeks.

### Construction of the vectors carrying the resistance genes

Amino acid sequences of the tunicamycin resistance protein (*tmr*B, GenBank P12921.4; Harada et al., 1988) and the *bsr* (GenBank P33967.1; Kobayashi et al., 1991) were retrieved from GenBank. The amino acid sequences were reverse translated with the online-tool “Reverse Translate” of the “Sequence Manipulation Suite” (SMS, http://www.bioinformatics.org/sms2/rev_trans.html) (Stothard, 2000). Recognition sites of popular commercially available restriction enzymes were then manually eliminated (the final gene sequences are shown in Data S1). We then checked the codon efficiency of the genes with a custom generated codon usage table for *P. tricornutum* (Table S1), prepared in the following way: The coding sequences (cds) of the *P. tricornutum* gene catalog Phatr3 (Bowler et al., 2008; Rastogi et al., 2018) were downloaded from ftp://ftp.ensemblgenomes.org/pub/protists/release-39/fasta/phaeodactylum_tricornutum/cds/ (file name Phaeodactylum_tricornutum.ASM15095v2.cds.all_.fa). The 12178 cds were then screened for sequence lengths that are divisible by three. If necessary, sequences were processed to a sequence length that is divisible by three with help of the custom perl script “divisible_by_three.pl” (https://bitbucket.org/grubio-/divisible_by_three). 12177 cds had lengths that were either divisible by three, or could be truncated to complete codons in reference to a start or a stop codon, resulting in a total of 18112998 base pairs (compared to 18113425 base pairs in the complete Phatr3 cds catalog). Sequences were then concatenated into a single sequence with the “Combine FASTA” tool of a locally installed version of SMS (Stothard, 2000), and the codon table was generated using the tool “Codon Usage” of SMS (Stothard, 2000).

The reverse translated sequences were synthesized and cloned into a standard plasmid by Eurofins Genomics (Ebersberg, Germany). The genes were amplified by PCR (primers 01 and 02 for *bsr* and 03 and 04 for *tmr*B, Table S2) using KapaHifi polymerase (Roche, Basel, Switzerland).

The plasmid pPha-T1 (Zaslavskaia et al., 2000) was used as a template for a deletion PCR (primers 05 and 06, Table S2) to remove the *Sh*Ble gene and to introduce recognition sites for the restriction enzymes *Mss*I and *Eco*RI. The resulting PCR product was ligated and delivered to *E. coli* XL1 Blue by electroporation, followed by selection on ampicillin plates. Plasmid preparation was done according to the manufacturer’s protocol using “QIAprep Spin Miniprep Kit” (QIAGEN, Hilden, Germany). The plasmid DNA was treated with the restriction enzyme *Mss*I (Thermo Fisher, Waltham, MA, USA) and ligated with the amplified resistance genes using T4 DNA ligase (Thermo Fisher, Waltham, MA, USA). These constructs were genetically transformed to *E. coli* XL1 Blue by electroporation. Resulting colonies were screened by PCR for correct orientation of the insert. Extracted plasmid DNAs of single colonies growing on ampicillin plates were sequenced (Microsynth Seqlab, Göttingen, Germany) and used for the experiments with *P. tricornutum*.

### Vector sequences and maps

Vector maps were generated with Geneious version 9 (Kearse et al., 2012). The sequences of the reverse translated resistance genes and of the whole plasmids can be found in Supplemental Information S1. The sequence of the pPTbsr vector is additionally available at GenBank (accession number MH541819) and the vector will be available at Addgene with the deposit number 117696.

### PCR test for presence of *bsr* gene

A small amount of blasticidin-S resistant colonies (transformed with pPTbsr) was scraped off the plates, dissolved in 100 μl of water and heated to 99 °C for 10 min to serve as template. The PCR mixture contained five μl of this template, 0.1 μl of Taq DNA Polymerase E (Genaxxon bioscience, Ulm, Germany), 2.5 μl of 10× buffer, 0.25 μl of each primer (01 and 02 for the whole gene, 07 and 08 for a shorter fragment; 10 mM, Table S2), 0.25 μl of dNTPs (10 mM), 17 μl of H_2_O. The PCRs were performed in a PCR cycler (ThermoCycler; Eppendorf, Hamburg, Germany) as described by the manufacturer of the DNA polymerase with annealing temperature of 54 °C and an extension time of 40 s.

### Cross activities of marker genes

Nine of the blasticidin-S-resistant *P. tricornutum* colonies, transformed with the plasmid “pPTbsr” and nine nourseothricin-resistant colonies created by transformation of wild-type *P. tricornutum* with the plasmid “pNat” (Zaslavskaia et al., 2000), as well as five of the Zeocin resistant “lhcf1” cell lines generated by Chu et al. (2016) as well as two TGS1 and two TGS2 cell lines generated by Huang, Río Bártulos & Kroth, (2016) were used for the detection of possible cross-resistances against the antibiotics conferred by one of the tested marker genes.

## Results and Discussion

To identify new antibiotics suitable for selection of *P. tricornutum*, we first established a workflow consisting of three main steps. In the first step, the adequate amount of an antibiotic was determined that leads to cell death in *P. tricornutum*. In the second step, potential resistance genes that conferred resistance against the respective antibiotics were tested in the target organism. Finally, the respective resistance proteins were checked for any cross activity with the two other antibiotics in use (Zeocin and nourseothricin), which is required for independent selection with combinations of the antibiotics.

### Determination of effective concentrations of the antibiotics

**Figure 1:**
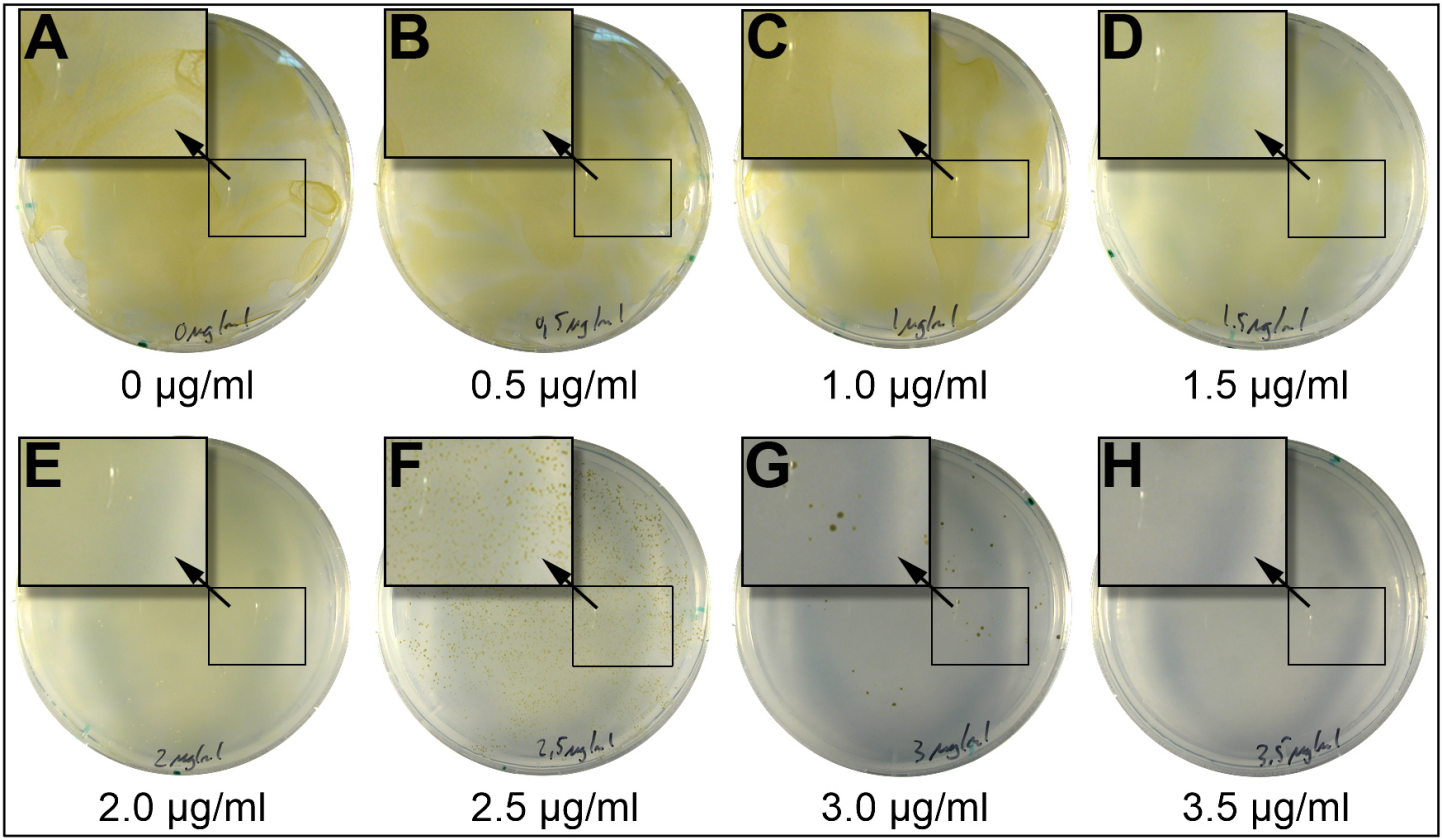
Incubation of *P. tricornutum* on plates containing different concentrations of blasticidin-S. 2.5 × 10^7^ *P. tricornutum* cells were spread on plates (25% salinity of seawater) and containing different concentrations of blasticidin-S. At concentrations between 0 and two μg/ml of blasticidin-S (A–E) we observed a lawn of cells. At 2.5 μg/ml (F) and 3.0 μg/ml (G) we observed scattered growth, and above 3.5 μg/ml (H) no growth was observed. Magnified areas in the picture are indicated by an arrow.

A classical way to determine the lethal concentration of an antibiotic is a dose-response experiment, in which cells are subjected to increasing amounts of an antibiotic to determine the minimal efficient antibiotic concentration that is needed to inhibit cell growth. Accordingly, we tested the efficiency of the antibiotics by cultivating *P. tricornutum* cells on plates containing different concentrations of antibiotics. According to previous publications, a lethal dose of blasticidin-S of about two μg/ml was reported for mammalian cells (Izumi et al., 1991), for tunicamycin the LD_50_ was reported between 0.1 and five μg/ml (Elbein, 1981), while voriconazole at 0.35 μg/ml impaired plant growth (Rozhon et al., 2013). For our experiments, we used concentrations ranges from higher to lower than these values (Table 1). We plated 2.5 × 10^7^ wild-type cells, and the growth of cells in each plate was determined after 3 and 5 weeks, classifying the results into “no growth”, “scattered growth”, or “diatom lawns” (Fig. 1; Table 1). Some antibiotics like G418 and nourseothricin are inhibited by high salt concentrations in marine media (Zaslavskaia et al., 2000). Blasticidin-S has been described by the manufacturer to be affected by the presence of salts, we therefore additionally tested a lower salt concentration in the medium (50% and 25% sea salt concentration). *P. tricornutum* is known to occur in places with highly variable growth conditions (Martino et al., 2007) and to tolerate a wide range of salt concentrations (Kräbs & Büchel, 2011).

**Table 1:** Determination of effective antibiotic concentrations.

Antibiotic	Salt concentration	Antibiotic concentration (μg/ml)	Results		Ind. Repl.
			3 Weeks	5 Weeks	
**Blasticidin-S**	1/2	2	Lawn	Lawn	6
	1/2	4, 6	Scattered	Scattered	6
	1/2	8, 10	No	No	6
	1/4	0.5, 1, 1.5, 2, 2.5	Lawn	Lawn	10
	1/4	3	Scattered	Scattered	10
	1/4	3.5	No	No	4
	1/4	4	No	No	24
	1/4	6	No	No	2
**Tunicamycin**	1/2	0.075, 0.15, 0.3, 0.6	Lawn	Lawn	1
	1/2	1.25, 2.5	Scattered	Scattered	1
	1/2	5, 10	No	Scattered	6
	1/4	5	No	No	6
	1/4	10	No	No	2
**Voriconazole**	1/2	0.4, 0.7, 1.1, 3.5, 7, 10.5, 35, 70, 105	Lawn	Lawn	1

**Note:**

2.5 × 10^7^ *P. tricornutum* cells were plated on each agar plate containing different concentrations of antibiotics. Tunicamycin and blasticidin-S were tested both on 50% and 25% sea salinity plates. Growth was checked after 3 and 5 weeks of incubation. “Ind. repl.” = independent replicates, “no” = no growth, “scattered” = growth in single colonies, “lawn” = lawn growth.

Voriconazole, an inhibitor of P450 in plants (Rozhon et al., 2013), did not show any effect on *P. tricornutum* growth even at 105 μg/ml, which is about 300-fold the concentration of effective doses for most land plants (Rozhon et al., 2013). Therefore, this antibiotic was not investigated further. Blasticidin-S is an inhibitor of protein synthesis binding to the P-site of the 60S ribosomal subunit (Garreau De Loubresse et al., 2014). Blasticidin-S inhibited the growth of *P. tricornutum* completely at a concentration of 3.5 μg/ml on plates with 25% salt and eight μg/ml on 50% salt plates (Table 1). In liquid 50% salt medium, blasticidin-S inhibited growth at a concentration of three μg/ml (Fig. S1). Tunicamycin, which acts as inhibitor of biosynthesis of peptidoglycan structures in prokaryotes and of *N*-glycosylation in eukaryotes (Takatsuki, Arima & Tamura, 1971; Wyszynski et al., 2012), inhibited the growth at concentrations of five μg/ml on 25% salt plates, and 10 μg/ml on 50% salt plates. However, in contrast to blasticidin-S, tunicamycin was not able to stop growth completely, as single colonies appeared after 5 weeks on the 50% salt plates.

### Functionality of the respective resistance genes

**Figure 2:**
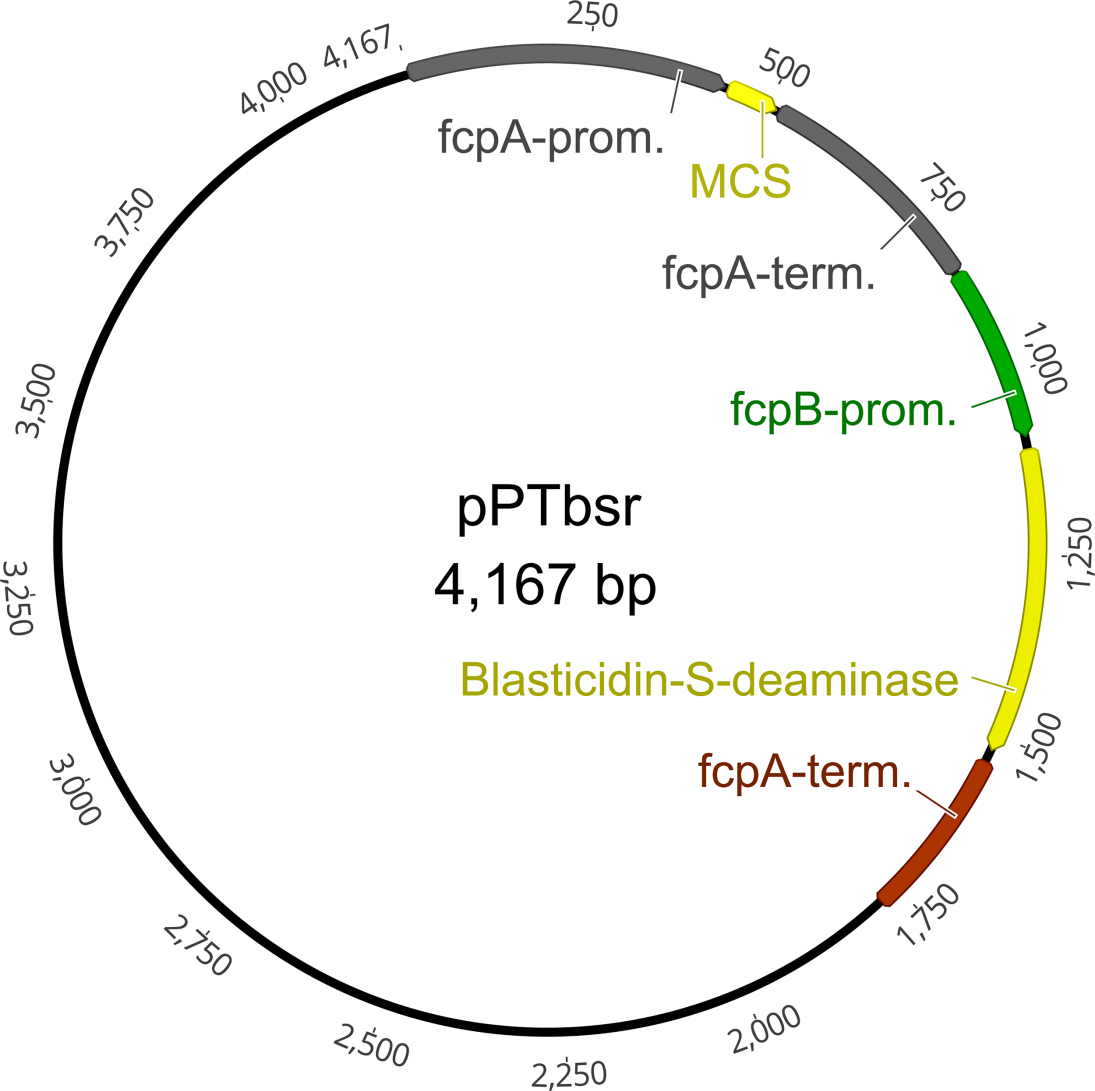
Plasmid map of the vector pPTbsr. The vector is based on pPha-T1 (derived from Zaslavskaia et al. (2000)) but includes the resistance gene blasticidin-S deaminase (*bsr*) instead of the Zeocin resistance cassette *Sh*Ble. MCS, multiple cloning site; fcpA/B, fucoxanthin-chlorophyll-binding protein A/B; prom, promoter; term, terminator.

In order to confer resistance against tunicamycin and blasticidin-S in *P. tricornutum*, we generated DNA fragments encoding the tunicamycin resistance protein (*tmr*B) (Harada et al., 1988) and the *bsr* (Endo et al., 1987), with codons suitable for *P. tricornutum* and manually eliminated recognition sites of popular commercially available restriction enzymes. We inserted the synthetic genes into the plasmid pPha-T1 (Zaslavskaia et al., 2000), replacing the Zeocin resistance cassette *Sh*Ble between the fcpB-promoter and the fcpA-terminator (Fig. 2, example for the *bsr* gene). *P. tricornutum* was genetically transformed with the constructs by particle bombardment. To test the antibiotic resistance, we plated the cells on selection plates containing four μg/ml (25% salt) or 10 μg/ml (50% salt) of blasticidin-S, and 10 μg/ml (50% salt) tunicamycin, respectively. For unknown reasons, we only obtained blasticidin-S-resistant colonies for the cells transformed with the *bsr* gene, while no tunicamycin-resistant colonies were obtained when we tested the *tmr*B gene using two independent attempts.

In total, we obtained 78 blasticidin-S-resistant colonies on four μg/ml, 25% salt medium in two independent transformations, plus 22 colonies on 10 μg/ml, 50% salt medium in one transformation approach. We re-plated each of the obtained colonies on fresh blasticidin-S plates, on which all of them continued to grow stably and were able to survive in liquid medium under selection pressure (Fig. S1). In more than one year that has passed since we obtained the first blasticidin-S cell lines, we did not observe a loss of resistance in any of the colonies. For further experiments we focused only on colonies that were selected on the 25% salt plates. These 78 colonies were checked by PCR for the presence of the *bsr* gene. We could amplify a PCR product in the expected size range from 75 of the 78 analyzed templates derived from the colonies, indicating the presence of the resistance gene (Fig. S2).

### Cross reactivity test

**Figure 3:**
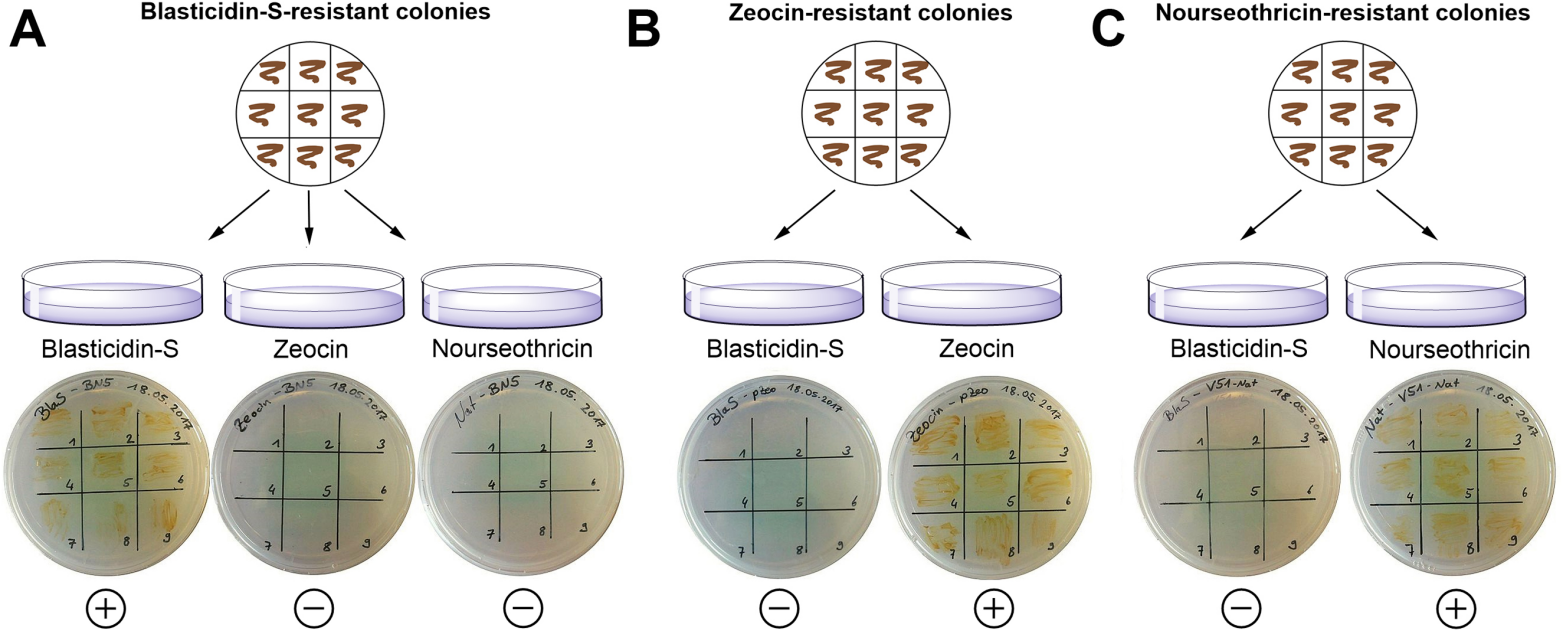
Exclusion of potential interference between different resistance genes using strains showing either Zeocin, nourseothricin or blasticidin-S resistance. (A) Nine blasticidin-S-resistant *P. tricornutum* colonies were spread on plates containing Zeocin or nourseothricin and on a control plate containing blasticidin-S. (B) Nine Zeocin-resistant colonies were spread on blasticidin-S plates and on control plates with Zeocin. (C) Nine nourseothricin-resistant colonies were spread on plates with blasticidin-S and on control plates with nourseothricin. The cells were able to survive only on the appropriate antibiotics. (+) = growth of the cells; (−) = no growth.

In order to perform successive transformations with different selection markers, we tested whether blasticidin-S might interfere with the usage of Zeocin- or nourseothricin-based screening protocols. We therefore plated nine of the blasticidin-S-resistant colonies on agar plates containing either Zeocin or nourseothricin (Fig. 3). Conversely, nine nourseothricin- or Zeocin-resistant colonies were plated on plates with four μg/ml blasticidin-S. As a control, the same colonies were inoculated on culture plates containing the antibiotic to which they show resistance. After 11 days of growth, colonies were detected only on plates on which cells were plated that had the respective antibiotic resistance (Fig. 3). This indicates that blasticidin-S selection can be used in combination with Zeocin- or nourseothricin-resistant cells. Such double resistant strains were already reported for the combination of Zeocin and nourseothricin (Serif et al., 2017). Especially the combination of blasticidin-S- and nourseothricin might be important for future applications, for instance to replace Zeocin in the TALEN vectors or for complementation experiments following gene knockouts. We co-transformed the plasmids pPTbsr and pPha-NR (without *Bsa*I) (Serif et al., 2017), which mediates nourseothricin resistance, leading to 29 resistant colonies on the 50% salt selection plates containing 10 μg/ml blasticidin-S and 150 μg/ml nourseothricin.

### Transferability of the results to other strains and diatom species

In this study we used *P. tricornutum* strain UTEX 646 (Pt4 in the nomenclature by Martino et al. (2007)) in all our experiments. Experience with other antibiotics like Zeocin/phleomycin shows that there is apparently no difference between different strains regarding their sensitivity (Apt, Grossman & Kroth-Pancic, 1996; Falciatore et al., 1999; Siaut et al., 2007; Tanaka et al., 2005). Furthermore, Martino et al. (2007) used the *Sh*Ble marker gene for ten different strains of *P. tricornutum*. We therefore expect that our results regarding blasticidin-S are directly transferable to other *P. tricornutum* strains.

In contrast to this, the sensitivity of different diatom species to the currently used antibiotics is quite variable (Huang & Daboussi, 2017) and although some antibiotics, after dose adjustment, can be used in several species of diatoms, marker genes are not generally transferrable between diatom species (Huang & Daboussi, 2017). It therefore remains to be shown if blasticidin-S is suitable for the selection of genetically transformed cell lines of other diatom species. For this, most likely adjustments of the promoters and antibiotic doses would be needed.

## Conclusions

Here we report a new and easy to handle system of antibiotic selection with blasticidin-S and the resistance gene *bsr* in *P. tricornutum*. Blasticidin-S works by blocking protein translation and therefore it is not expected to affect the genomic DNA of transformed cells. Especially for reverse genetics studies via genome editing, when phenotypes should be determined based on cell-linages with as less off-target genetic changes as possible, utilization of this antibiotic is a major improvement. Additionally, blasticidin-S selection can be used in combination with nourseothricin- and potentially also with Zeocin-resistant cells. This extension of the genetic toolbox will be very useful for further molecular characterization or biotechnological application of the model diatom *P. tricornutum* and possibly for other species of diatoms.

## Supplemental Information

10.7717/peerj.5884/supp-1 Supplemental Information 1 Sequences of the resistance genes *bsr, tmr*B and the complete plasmids pPTbsr and pPTtmrB.

10.7717/peerj.5884/supp-2 Supplemental Information 2 Codon usage in *P. tricornutum*. The table shows the codon frequency for each codon in *P. tricornutum*. “Number” is the total count for each codon within all protein coding genes (see text for details), “/1000” is the frequency of a codon (per 1000 total codons), “Fraction” is the relative proportion of a single codon within the synonymous codons.

10.7717/peerj.5884/supp-3 Supplemental Information 3 Primer sequences used in this study.

10.7717/peerj.5884/supp-4 Supplemental Information 4 Determination of lethal concentration of blasticidin-S in liquid 50% salt medium. *P. tricornutum* was inoculated with 10^5^ cells/ml in liquid 50% salt medium with different concentrations of blasticidin-S. The growth was determined six days after inoculation. **(A)** Wild type cells in 1 μg/ml blasticidin-S **(B)** Wild type cells in 2 μg/ml blasticidin-S **(C)** wild type cells in 3 μg/ml blasticidin-S **(D)** Wild type cells in 4 μg/ml blasticidin-S **(E)** Wild type cells in 5 μg/ml blasticidin-S **(F)** Resistant cells in 2 μg/ml blasticidin-S **(G)** Resistant cells in 3 μg/ml blasticidin-S **(H)** Resistant cells in 4 μg/ml blasticidin-S. Growth of the wild-type cells was inhibited at a concentration of 3 μg/ml or higher, while the pPTbsr transformed cell line survived 4 μg/ml.

10.7717/peerj.5884/supp-5 Supplemental Information 5 Agarose-gels with PCR products using each blasticidin-S resistant colony as template. **(A)** Primers for amplification of the whole *bsr* gene (primers 01 and 02; expected length of 423 base pairs) or **(B)** for an internal part (primers 07 and 08; expected lenght of 216 base pairs) were used. As negative control, wild-type cells were used as template. “1kb” = O&GeneRuler 1 kb DNA-ladder (Thermo Fisher, Waltham, MA, USA); “50bp” = O&GeneRuler 50 bp DNA-ladder (Thermo Fisher, Waltham, MA, USA); “P” = positive control (positive colonies as PCR Template); “C” = Control (plasmid-DNA (pPTbsr) as PCR template); “bp” = base pairs.

## Additional Information and Declarations

### DNA Deposition

The following information was supplied regarding the deposition of DNA sequences: Sequences were uploaded to GenBank (accession number MH541819) and can also be found in Supplemental Dataset 1.

### Data Availability

The following information was supplied regarding data availability: Data can be found at GenBank, accession number GenBank: MH541819
