# Supplementary material for: Blasticidin-S deaminase, a new selection marker for genetic transformation of the diatom Phaeodactylum tricornutum
Source: PeerJ. 2018 Nov 14;6:e5884. doi: 10.7717/peerj.5884 (PMC6250098; doi:10.7717/peerj.5884)
Supplement: Supplemental Information 2 — The table shows the codon frequency for each codon in P. tricornutum. “Number” is the total count for each codon within all protein coding genes (see text for details), “/1000” is the frequency of a codon (per 1000 total codons), “Fraction” is the relative proportion of a single codon within the synonymous codons. [file peerj-06-5884-s002.docx]

AmAcid Codon Number /1000 Fraction ..

Ala GCG 113760.00 18.84 0.22

Ala GCA 109945.00 18.21 0.21

Ala GCT 126124.00 20.89 0.25

Ala GCC 162958.00 26.99 0.32

Cys TGT 46540.00 7.71 0.50

Cys TGC 47468.00 7.86 0.50

Asp GAT 178709.00 29.60 0.50

Asp GAC 178015.00 29.48 0.50

Glu GAG 135023.00 22.36 0.37

Glu GAA 233047.00 38.60 0.63

Phe TTT 132726.00 21.98 0.60

Phe TTC 87868.00 14.55 0.40

Gly GGG 59473.00 9.85 0.16

Gly GGA 118931.00 19.70 0.31

Gly GGT 107081.00 17.74 0.28

Gly GGC 98202.00 16.27 0.26

His CAT 65895.00 10.91 0.44

His CAC 83993.00 13.91 0.56

Ile ATA 42731.00 7.08 0.16

Ile ATT 136081.00 22.54 0.51

Ile ATC 89723.00 14.86 0.33

Lys AAG 143131.00 23.71 0.51

Lys AAA 139750.00 23.15 0.49

Leu TTG 161073.00 26.68 0.29

Leu TTA 46951.00 7.78 0.08

Leu CTG 99537.00 16.49 0.18

Leu CTA 54915.00 9.10 0.10

Leu CTT 92930.00 15.39 0.17

Leu CTC 106382.00 17.62 0.19

Met ATG 134586.00 22.29 1.00

Asn AAT 109807.00 18.19 0.48

Asn AAC 119160.00 19.74 0.52

Pro CCG 85988.00 14.24 0.27

Pro CCA 78344.00 12.98 0.25

Pro CCT 70703.00 11.71 0.22

Pro CCC 82045.00 13.59 0.26

Gln CAG 107292.00 17.77 0.44

Gln CAA 139180.00 23.05 0.56

Arg AGG 26822.00 4.44 0.07

Arg AGA 38528.00 6.38 0.10

Arg CGG 62204.00 10.30 0.17

Arg CGA 84441.00 13.99 0.23

Arg CGT 78113.00 12.94 0.21

Arg CGC 82911.00 13.73 0.22

Ser AGT 76559.00 12.68 0.15

Ser AGC 75294.00 12.47 0.15

Ser TCG 109251.00 18.10 0.21

Ser TCA 67086.00 11.11 0.13

Ser TCT 80976.00 13.41 0.16

Ser TCC 104539.00 17.31 0.20

Thr ACG 104530.00 17.31 0.28

Thr ACA 84777.00 14.04 0.23

Thr ACT 79765.00 13.21 0.21

Thr ACC 102923.00 17.05 0.28

Val GTG 107548.00 17.81 0.26

Val GTA 69733.00 11.55 0.17

Val GTT 108552.00 17.98 0.26

Val GTC 124239.00 20.58 0.30

Trp TGG 79735.00 13.21 1.00

Tyr TAT 56061.00 9.29 0.37

Tyr TAC 94868.00 15.71 0.63

End TGA 3424.00 0.57 0.28

End TAG 3939.00 0.65 0.33

End TAA 4710.00 0.78 0.39
