# Supplementary material for: Blasticidin-S deaminase, a new selection marker for genetic transformation of the diatom Phaeodactylum tricornutum
Source: PeerJ. 2018 Nov 14;6:e5884. doi: 10.7717/peerj.5884 (PMC6250098; doi:10.7717/peerj.5884)
Supplement: Supplemental Information 3 [file peerj-06-5884-s003.docx]

| **Primer** | **Sequence 5’->3’** | **Target** |
| --- | --- | --- |
| **01** | ATGAAAACGTTTAATATCTCGC | Amplification + check for presence of *bsr* (forward) |
| **02** | TCAATTTCGCGTGTATTTAAG | Amplification + check for presence of *bsr* (reverse) |
| **03** | ATGATTATTTGGATTAATGGAGC | Amplification of *tmr*B (forward) |
| **04** | TCATTTAATTCGAATATGATTAAGTTTG | Amplification of *tmr*B (reverse) |
| **05** | GTTTAAACCAGGACTGAACCTTCC | Deletion of *Sh*Ble (reverse) |
| **06** | CAATTGTCTTGACATCTGGCAAC | Deletion of *Sh*Ble (forward) |
| **07** | GTCGGAGCAGCAATTCG | Check for presence of *bsr* (forward) |
| **08** | GCACATTCCGCATGG | Check for presence of *bsr* (reverse) |
