# Supplementary figures and images for: Blasticidin-S deaminase, a new selection marker for genetic transformation of the diatom Phaeodactylum tricornutum
Source: PeerJ. 2018 Nov 14;6:e5884. doi: 10.7717/peerj.5884 (PMC6250098; doi:10.7717/peerj.5884)

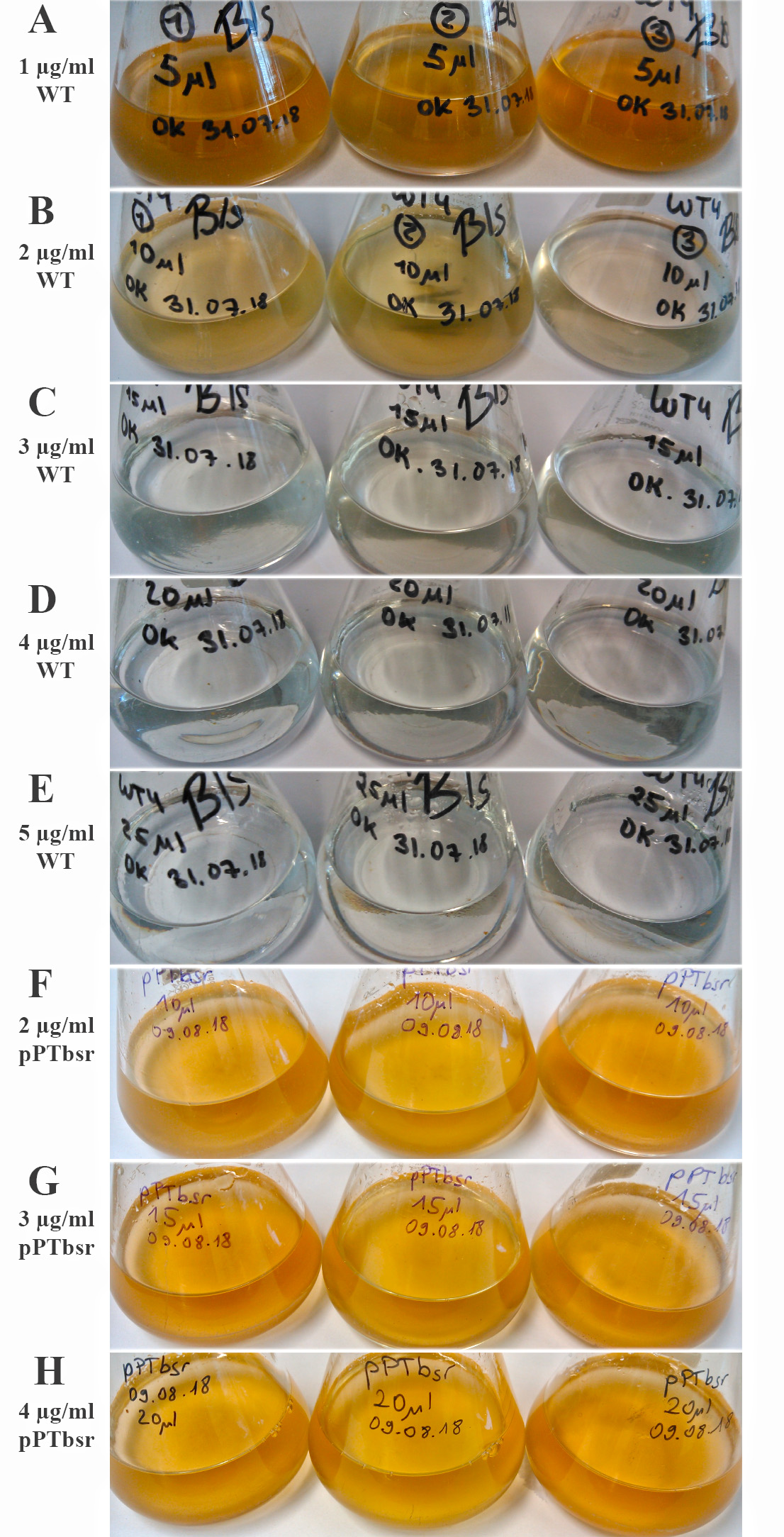

Supplement: Supplemental Information 4 — P. tricornutum was inoculated with 105 cells/ml in liquid 50% salt medium with different concentrations of blasticidin-S. The growth was determined six days after inoculation. (A) Wild type cells in 1 μg/ml blasticidin-S (B) Wild type cells in 2 μg/ml blasticidin-S (C) wild type cells in 3 μg/ml blasticidin-S (D) Wild type cells in 4 μg/ml blasticidin-S (E) Wild type cells in 5 μg/ml blasticidin-S (F) Resistant cells in 2 μg/ml blasticidin-S (G) Resistant cells in 3 μg/ml blasticidin-S (H) Resistant cells in 4 μg/ml blasticidin-S. Growth of the wild-type cells was inhibited at a concentration of 3 μg/ml or higher, while the pPTbsr transformed cell line survived 4 μg/ml. [file peerj-06-5884-s004.jpg]

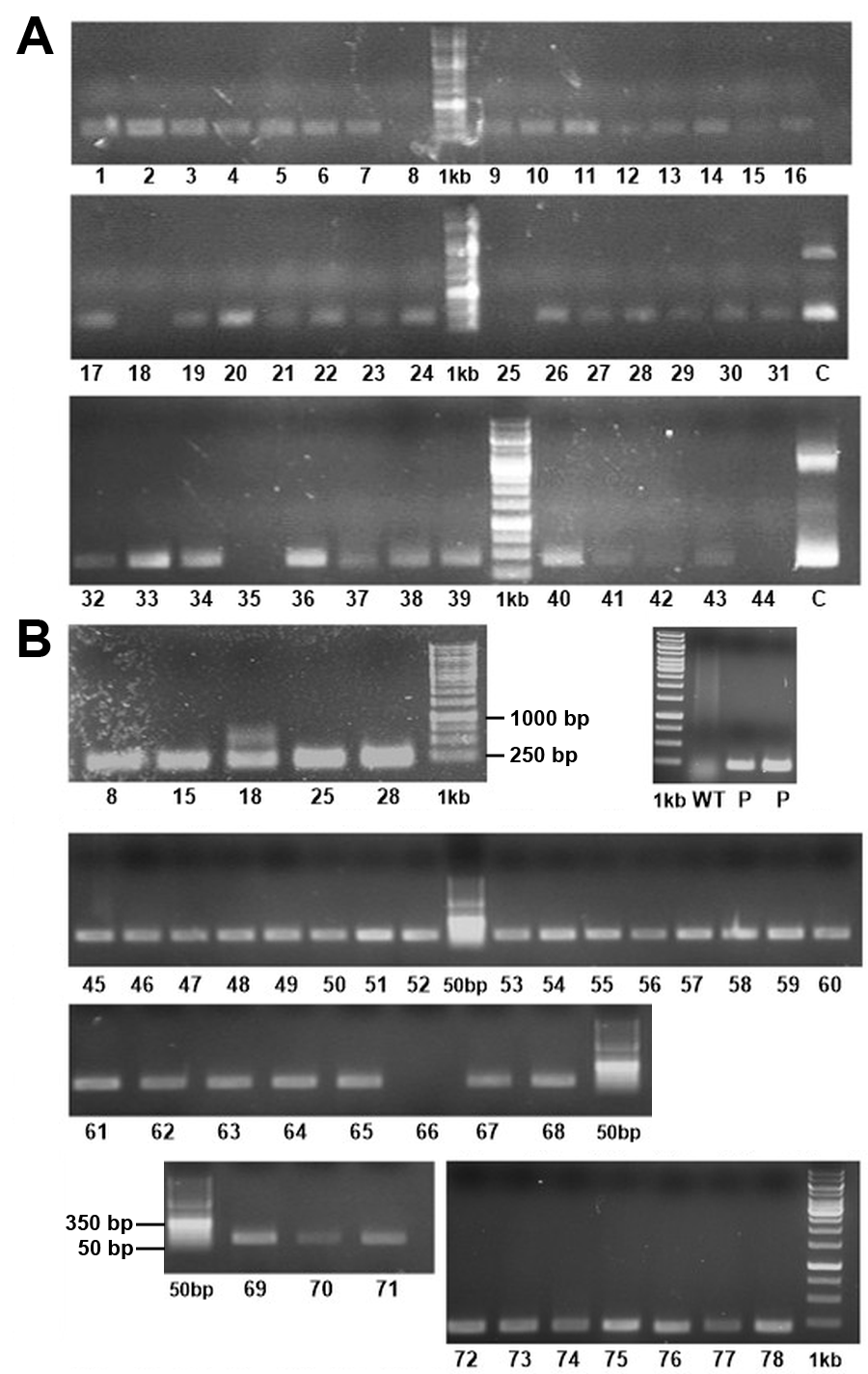

Supplement: Supplemental Information 5 — (A) Primers for amplification of the whole bsr gene (primers 01 and 02; expected length of 423 base pairs) or (B) for an internal part (primers 07 and 08; expected lenght of 216 base pairs) were used. As negative control, wild-type cells were used as template. “1kb” = O&GeneRuler 1 kb DNA-ladder (Thermo Fisher, Waltham, MA, USA); “50bp” = O&GeneRuler 50 bp DNA-ladder (Thermo Fisher, Waltham, MA, USA); “P” = positive control (positive colonies as PCR Template); “C” = Control (plasmid-DNA (pPTbsr) as PCR template); “bp” = base pairs. [file peerj-06-5884-s005.png]
